# Health Insurance Portability and Accountability Act Liability in the Age of Generative Artificial Intelligence

**DOI:** 10.1016/j.acepjo.2025.100317

**Published:** 2026-01-22

**Authors:** Dave Schoolcraft, Andrew C. Meltzer, Rohit Sangal, Aisha T. Terry, Katherine Robertson, Daniel Buckland, Sakib Motalib, Nicholas Genes, Rade Vukmir, Tayab Waseem, ACEP AI TASK FORCE

**Affiliations:** 1 Ogden Murphy Wallace, Seattle, WA; 2 Department of Emergency Medicine, The George Washington University School of Medicine and Health Sciences, Washington, DC; 3 Department of Emergency Medicine, Yale School of Medicine, New Haven, CT; 4 Department of Emergency Medicine, University of Wisconsin - Madison, Madison, WI; 5 Department of Emergency Medicine, Inova Fairfax Hospital, Falls Church, VA; 6 Ronald O. Perelman Department of Emergency Medicine, NYU Grossman School of Medicine, New York, NY; 7 Clinical Emergency Medicine, Drexel University, Philadelphia, PA

**Keywords:** HIPPA, AI, generative AI, liability, BAA, risk management

## Abstract

As artificial intelligence tools become increasingly integrated into emergency department workflows, healthcare providers face a growing risk of legal liability stemming from improper use, particularly with respect to data privacy and Health Insurance Portability and Accountability Act (HIPAA) compliance. This article explores a realistic clinical scenario in which an emergency physician inadvertently violates HIPAA using a publicly available AI tool, such as ChatGPT, Gemini, Llama, and Grok, without a valid Business Associate Agreement in place.

We review the legal framework of the HIPAA Privacy, Security, and Breach Notification Rules and delineate the respective liabilities of healthcare institutions and individual clinicians. Key distinctions are made between incidental, accidental, and unauthorized disclosures of protected health information, and we provide clear guidance on post-breach mitigation steps. The article also discusses the statistical likelihood of protected health information reidentification or reproduction by AI models and outlines risks associated with state-level data protection laws.

Ultimately, we offer practical recommendations for physicians seeking to leverage AI responsibly in clinical care, including verifying institutional Business Associate Agreements, understanding platform-specific privacy policies, and consulting with privacy officers before entering any patient data. As AI rapidly evolves, clinicians must remain vigilant in safeguarding patient information to avoid legal exposure and uphold ethical standards of care.

## 1 Introduction

Artificial intelligence (AI) tools are rapidly entering the clinical workspace, promising to improve efficiency, streamline documentation, and augment the decision-making process across a wide range of medical settings, including the emergency department (ED). Among these tools, large language models (LLMs) such as ChatGPT, Gemini, Llama (Meta AI), and Grok are increasingly accessible and intuitive, making them attractive options for physicians seeking real-time support in tasks such as patient communication, diagnostic guidance, or discharge planning.

The rapid adoption of AI in healthcare has created a critical regulatory compliance gap. Although a significant majority of US physicians (66%) now report actively using AI tools in their practice, a key measure of institutional protection remains woefully low: only about 23% of health systems report having Business Associate Agreements (BAAs) in place to ensure Health Insurance Portability and Accountability Act (HIPAA) compliance when deploying third-party AI solutions.^1^^,^^2^ This striking imbalance highlights that what may appear to be “isolated hypotheticals” are, in reality, daily occurrences across US hospitals and clinics, underscoring the urgency for clear legal and operational guidance.

However, the legal and regulatory landscape surrounding AI use in clinical care remains underdeveloped.^3^ In particular, the use of AI tools that are not explicitly approved for handling protected health information (PHI) may expose physicians and healthcare institutions to significant liability under the HIPAA and relevant state-level data protection laws.^3^^,^^4^ Even well-intentioned use of an AI application, such as generating discharge instructions using a chatbot, may result in unauthorized disclosure of PHI if institutional safeguards, such as a BAA, are not in place.^3^, ^4^, ^5^

This article explores situations in which a physician’s casual use of an AI tool leads to a potential HIPAA violation. We review the key provisions of HIPAA related to AI use, assess institutional and individual liability, describe the risk of PHI exposure through LLMs, and offer actionable recommendations for ED providers navigating the adoption of AI. As the use of AI tools continues to expand in emergency medicine, understanding the legal boundaries of their application is critical to safeguarding both patients and providers.

### 1.1 HIPAA Framework and AI Use

The HIPAA establishes national standards for the protection of patient health information. AI tools such as LLMs challenge existing HIPAA frameworks, particularly when used without appropriate institutional safeguards. Understanding HIPAA’s three foundational rules is essential for assessing the legality of AI use in clinical settings:

#### 1.1.1 The Privacy Rule

The HIPAA Privacy Rule (45 CFR §§ 164.500–534) protects identifiable health information by regulating its use and disclosure by covered entities and their business associates. PHI is any element that can reasonably identify a patient, including any 18 specified health-related data identifiers (Table 1) when linked with clinical data. Although PHI may be used for treatment, payment, and healthcare operations without explicit patient consent, any disclosure to a third-party vendor must occur under a valid BAA.^3^ The BAA contractually extends HIPAA’s privacy and security obligations to the third-party vendor.

**Table 1:** Protected health information (PHI) identifiers under HIPAA.

#	Identifier	Description
1	Names	Full names or initials.
2	Geographic subdivisions	All elements smaller than a state (eg, street address, city, county, precinct, zip code, and their equivalent geocodes). ZIP code exception: The first three digits may be used only if the geographic unit contains >20,000 people; otherwise, replace with 000.
3	Dates	All elements of dates (except year) directly related to an individual, including birth date, admission date, discharge date, date of death, and all ages over 89 and all elements of dates (including year) indicative of such age.
4	Telephone numbers	Any part of a phone number.
5	Fax numbers	Any part of a fax number.
6	Electronic mail addresses	Email addresses.
7	Social security numbers (SSN)	Any part of an SSN.
8	Medical record numbers (MRN)	Any part of a medical record number.
9	Health Plan Beneficiary Numbers	Any part of a health plan member’s number.
10	Account numbers	Any part of an account number.
11	Certificate/license numbers	Any part of a certificate or license number.
12	Vehicle identifiers	Including serial numbers, license plate numbers.
13	Device identifiers and serial Numbers	Any part of a device identifier or serial number.
14	Web universal resource Locators (URLs)	Web URLs.
15	Internet protocol (IP) address Numbers	IP addresses.
16	Biometric identifiers	Including finger and voice prints.
17	Full-face photographic images	And any comparable images.
18	Any other unique identifying Number, characteristic, or code	Unless otherwise permitted by the HIPAA rules.

HIPAA, Health Insurance Portability and Accountability Act; MRN, medical record numbers; PHI, protected health information; SSN, social security numbers.

#### 1.1.2 The Security Rule

The Security Rule (45 CFR § 164 Subpart C) outlines administrative, technical, and physical safeguards required to protect electronic PHI (ePHI). It mandates risk assessments, access controls, secure data transmission protocols, and workforce training to ensure the confidentiality, integrity, and availability of PHI. AI tools that process patient data must meet these standards, especially if integrated into electronic medical record systems or clinical workflows.^6^^,^^7^

#### 1.1.3 The Breach Notification Rule

Under the Breach Notification Rule (45 CFR §§ 164.400–414), any unauthorized use or disclosure of unsecured PHI constitutes a breach unless a risk assessment demonstrates a low probability of compromise. Factors considered include the nature of the data, the recipient, whether the PHI was viewed, and the extent of mitigation. Reportable breaches involving more than 500 individuals must be disclosed to the US Department of Health and Human Services within 60 days and may trigger media notification requirements.^3^, ^4^, ^5^, ^6^, ^7^

Without a BAA in place, even a brief or well-intentioned interaction with an AI tool, such as inputting PHI into a chatbot to generate discharge instructions, can qualify as an unauthorized disclosure. This is true regardless of whether the data are stored or later accessed by the AI provider. Importantly, imaging data or multimedia content may carry embedded identifiers, further increasing the likelihood of a breach. As such, no AI tool should be used with PHI unless reviewed and approved by the institution’s legal or compliance team, and formal agreements are in place.

### 2.1 Legal Liability in AI-Driven Clinical Use

When a physician uses an AI tool without appropriate institutional safeguards, such as a BAA, the disclosure of patient data may constitute a HIPAA violation, even if the intent was to improve care. Understanding the scope of liability requires distinguishing between the responsibilities of healthcare institutions and those of individual providers.

### 2.2 Covered Entity Liability

Hospitals, clinics, and health systems are designated as “covered entities” under HIPAA and are primarily responsible for ensuring compliance with all privacy and security rules. This includes vetting any third-party vendor that handles PHI, ensuring appropriate data protections, and executing BAAs with such vendors. When a provider uses an AI tool without a valid BAA, and PHI is disclosed, the institution may be held accountable for the breach. Depending on the scope and nature of the violation, penalties may include substantial fines, corrective action plans, and increased regulatory oversight.^3^^,^^5^

### 2.3 Individual Provider Liability

While individual clinicians are less likely to face direct civil penalties under HIPAA, they remain accountable under institutional policies. Employers are required to impose sanctions for unauthorized disclosures, which may include retraining, formal warnings, suspension, or termination. Although criminal liability under HIPAA is theoretically possible, it is generally reserved for cases of willful misconduct, fraud, or intentional misuse of PHI for personal gain. In the case of a physician using an AI tool in good faith but without proper institutional authorization, disciplinary action is likely to remain administrative rather than legal.^3^

### 2.4 Shared Responsibilities and Risk

Importantly, legal risk is not confined to federal HIPAA enforcement. Depending on the jurisdiction, state privacy laws may impose additional liability for negligent handling of sensitive information. Some states allow patients to bring tort claims against providers or institutions for breaches of confidentiality, particularly when the breach results in demonstrable harm. As AI tools evolve and are increasingly embedded into clinical decision-making, the risk of attribution errors, incorrect outputs, or inappropriate data sharing will continue to grow, making legal vigilance essential at both the individual and system level.^3^^,^^7^

### 2.5 Mitigation and Risk Management

Once a potential breach involving AI and PHI is identified, immediate action is essential to contain the damage and meet regulatory obligations. Effective mitigation involves not only timely reporting and institutional response but also broader strategies to reduce future risk.

#### 2.5.1 Immediate Response After a Suspected Breach

If a clinician realizes that PHI was disclosed to an AI tool lacking a BAA, the first step should be to notify the institution’s privacy officer, compliance officer, and/or legal department. Ideally, the report should be reported through an established risk management process to minimize discoverability if possible. This report should include details such as the time period of disclosure, the type of data disclosed, the AI tool used, and the method of transmission. The institution is then responsible for conducting a risk assessment to determine whether the disclosure constitutes a reportable breach under HIPAA’s Breach Notification Rule.^5^^,^^6^

During this process, clinicians may be asked to assist in documenting the event, identifying the scope of information affected, and supporting patient notification or remediation efforts, if necessary. Prompt action can reduce legal liability and demonstrate good-faith compliance with regulatory expectations.

#### 2.5.2 Technical and Administrative Safeguards

Institutions can proactively reduce risk by developing clear policies on AI use, including

• **Restricting PHI input** into any AI tool unless a BAA is in place.
• **Requiring formal vetting** of any third-party software that interacts with patient data.
• **Implementing access controls** to limit which personnel can experiment with AI tools.
• **Disabling data retention** and training features on any approved AI tools.
• **Conducting regular staff education** on HIPAA-compliant technology use.

These safeguards align with HIPAA’s Security Rule requirements for managing ongoing and emerging risks in electronic health information systems.

#### 2.5.3 Role of the Individual Clinician

Physicians should adopt a risk-aware mindset when interacting with AI platforms. Before using any AI tool, even for routine documentation support, they should confirm whether the tool is institutionally approved and whether PHI input is permitted. Clinicians should avoid inputting identifiable information, including patient names, dates of birth, or medical record numbers, into any unapproved system. In addition, users should be cautious with imaging or multimedia files that may contain embedded identifiers, even if superficially deidentified. They should also apply the “minimum-necessary” principle: sharing only the data elements required for the task.

It is also a recommendation to document in the chart whenever AI output, as with other clinical tools, materially informs care. If an LLM-generated draft is inserted into the note, the clinician must review it for factual accuracy and sign it as their own work, just as they would with text generated by a scribe or content-importing technology from the EHR (electronic health record).

If the use of an AI tool is necessary for work, clinicians should seek guidance from institutional privacy officers regarding proper usage protocols and available privacy controls, such as disabling chat history or using enterprise versions of AI tools that offer enhanced protections.

#### 2.5.4 Institutional Culture and Preparedness

Finally, mitigation is most effective when part of a broader culture of digital ethics and legal compliance. Emergency departments, which are often early adopters of technology, should be particularly vigilant. Hospitals should foster an environment where staff feel safe reporting potential violations and where mitigation is seen as a shared responsibility rather than a punitive process. Periodic audits, updated training modules, and interdisciplinary oversight committees can help ensure that innovation does not outpace compliance.

### 2.6 Understanding Data Exposure in AI Tools

Even when unintended, entering PHI into a publicly available AI tool, such as an LLM, can result in meaningful data exposure. Unlike traditional search engines, which primarily log queries for analytics, AI tools may store, process, and even retain user input for model training, particularly when privacy controls are not activated. Understanding how AI platforms handle data and the statistical risk of inadvertent disclosure is critical for physicians exploring these technologies.^8^, ^9^, ^10^

#### 2.5.1 How AI Platforms Handle Input Data

Most commercial AI tools are designed to improve performance through user interactions. Unless privacy settings are modified or enterprise safeguards are in place, user input may be logged and stored. In some cases, such input may contribute to model training. This differs from a search engine, where queries are typically logged for analytics but not incorporated into generative algorithms.

In its default configuration, these LLMs may store inputs unless chat history is disabled, or the user is operating under a privacy-enhanced enterprise account. Importantly, even if a tool claims not to retain or reproduce exact text, the presence of unique or identifying PHI increases the statistical likelihood of model memorization and potential reproduction.^8^, ^9^, ^10^

#### 2.5.2 Memorization and Reproduction Risks

Although AI models are not explicitly designed to memorize and repeat exact input, studies have shown that LLMs can sometimes reproduce sensitive information, particularly when^8^, ^9^, ^10^

• The data are highly specific or unusual (eg, exact names, dates, or rare conditions).
• The input contains unique phrasing.
• The same data are entered repeatedly.
• The system lacks proper privacy safeguards.

#### 2.5.3 Risk Comparisons: AI Versus Traditional Tools

A side-by-side risk comparison helps contextualize exposure with the higher end of the likelihood used. Although the absolute risk of data reproduction by large language models remains low, it is useful to contextualize how AI-driven tools differ from traditional digital systems in their mechanisms of exposure. The table below (Table 2) provides an illustrative comparison to help emergency physicians conceptualize these differences.^8^, ^9^, ^10^ Importantly, even when the absolute risk is low, any disclosure of PHI to a non-HIPAA-compliant AI system constitutes a violation under the law’s strict liability framework

**Table 2:** Relative mechanisms of PHI exposure: AI tools versus traditional digital platforms.^1^

Scenario	Estimated likelihood of PHI exposure	Primary risk mechanism
Google search (normal use)	∼0.01%	Requires hacking or account compromise
AI tool (eg, ChatGPT)-typical prompt	∼0.01% to 0.1%	Transient model retention
AI tool-specific and unique input	∼0.5% to 1%	Memorization and potential reproduction
AI tool-repeated, detailed, self-identifying input	∼1% to 3%	Significant memorization risk

PHI, protected health information.

This table provides an illustrative, nonquantitative comparison of the relative mechanisms and likelihoods of PHI exposure across commonly used technologies. Estimates are derived from publicly available documentation and published analyses on model memorization.^8^, ^9^, ^10^ Values are approximate and intended solely for educational context to help physicians conceptualize relative risk, not as formal probabilistic modeling. The specific percentages cited in this report are our own extrapolated estimates. This is because direct, empirically verified rates from leading AI developers are not publicly available. This cautious estimation is grounded in the collective understanding from all available research, which consistently demonstrates LLM’s inherent memorization capabilities and their propensity to reproduce sensitive data, especially with unique or repeated inputs. Although studies on adversarial attacks show higher rates of data exposure under intentional exploitation, our figures represent a deliberate overestimation of risk for typical, nonmalicious use, ensuring a conservative approach to patient data privacy.

#### 2.5.4 What This Means for Physicians

AI tools may give the illusion of privacy, but unless operating under a valid BAA with strict data-use policies, entering PHI into these systems can result in legal exposure. Even if the probability of patient reidentification or model memorization is low, the act of disclosure itself, unauthorized and undocumented, may still constitute a HIPAA violation.^3^, ^4^, ^5^, ^6^, ^7^

Clinicians should be aware that disabling chat history or deleting past conversations in AI platforms may reduce future exposure risk but **does not retroactively legalize** the initial disclosure. As with EHR documentation, the metadata trail may persist. Only institutionally approved tools with documented compliance protocols should be used in patient care contexts.

### 2.6 Legal Landscape and Recommendations

The legal framework governing the use of AI in clinical care remains incomplete and evolving. Although federal regulations such as HIPAA form the backbone of patient privacy protections, state laws are increasingly influential in shaping provider liability. For physicians, this fragmented regulatory environment presents a high-stakes challenge: how to innovate responsibly without crossing legal boundaries.^3^^,^^7^^,^^11^

#### 2.6.1 Beyond HIPAA: State-Level Risk

Several states impose additional statutory or common-law duties regarding data privacy. For example, Washington State allows for tort claims against providers who negligently disclose sensitive health data, even if the disclosure does not meet HIPAA’s federal threshold for a reportable breach. Other states, such as California and New York, have comprehensive consumer data protection laws that could apply to medical data handled outside of traditional health-system channels. Certain categories of information, such as reproductive health, mental health, and substance use data, may carry additional state-level protections, further increasing liability if disclosed through AI tools without proper authorization.^11^

#### 2.6.2 Institutional Responsibilities

Healthcare systems must take proactive steps to reduce liability exposure and establish clear policies regarding AI tool use. Recommended institutional actions include the following:

• Executing **BAAs** with any AI vendors that may handle PHI.
• Providing **staff training** on the legal and ethical limitations of AI use in clinical care.
• Requiring **pre-approval of AI tools** prior to their use in documentation, communication, or decision support.
• Establishing **privacy-focused configurations**, such as disabling chat history, in approved tools.^12^
• Institutions should consider offering HIPAA-compliant, enterprise-grade AI tools to provide clinicians with safe, approved alternatives and reduce the risk of “shadow AI” use.

#### 2.6.3 Recommendations for Physicians

For individual clinicians, the safest path is caution. While AI offers meaningful benefits, especially in time-pressured emergency settings, those advantages must not come at the cost of patient privacy or professional accountability. Providers should:

• **Verify that a BAA is in place** between the institution and any AI tool before inputting patient data. Although questions have emerged about whether individual clinicians can or should enter into separate BAAs with AI vendors, this issue remains legally complex and context-dependent. A full analysis is beyond the scope of this article. Importantly, even if a clinician were to sign an individual BAA, most hospital and health-system policies still require institutional approval before any external digital tool may be used with PHI. In practice, this means that the presence of a BAA alone, whether at the individual or institutional level, does not automatically authorize AI use in clinical care.
• **Consult with the privacy or compliance officer** if unsure whether an AI tool is approved for use.
• **Avoid entering identifiable information** (eg, names, dates of birth, or unique diagnoses) into AI systems without institutional guidance.
• **Adjust platform settings** (eg, disable chat history, use enterprise versions) where possible to minimize retention.
• **Document all clinical decisions** independently of the AI tool, especially if AI output informs care recommendations.

## 3 Conclusion

AI tools are poised to transform emergency medicine, but only if integrated with careful attention to legal, ethical, and regulatory safeguards to protect patient information. Physicians must recognize that using AI to process or generate information involving PHI carries very real liability. A failure to align with HIPAA federal statutes and additional layered state-specific laws may result in disciplinary action, institutional fines, or legal liability. By staying informed, engaging institutional support, and maintaining transparency in technology use, providers can harness the power of AI without compromising patient privacy or professional integrity.

## Funding and Support

By *JACEP Open* policy, all authors are required to disclose any and all commercial, financial, and other relationships in any way related to the subject of this article as per ICMJE conflict of interest guidelines (see www.icmje.org). The authors have stated that no such relationships exist.

## Conflict of Interest Statement

All authors have affirmed they have no conflicts of interest to declare.
